# Impact of patient–provider gender concordance on mental health outcomes: scoping review

**DOI:** 10.1192/bjo.2026.12036

**Published:** 2026-07-17

**Authors:** James Lanni, Jason Denoncourt, Becky Baltich Nelson, Rebecca Drouhard, Stephany Eierle

**Affiliations:** University of Massachusetts Chan Medical School, Worcester, Massachusetts, USA; Lamar Soutter Library, University of Massachusetts Chan Medical School, Worcester, Massachusetts, USA; Primary Care Psychiatry, Family Health Center of Worcester, Worcester, Massachusetts, USA

**Keywords:** Gender matching, gender concordance, mental health, therapeutic alliance, psychotherapy

## Abstract

**Background:**

Mental health concerns are common. Despite this, evidence on the impact of patient–provider gender concordance on mental health outcomes remains limited.

**Aims:**

This review aims to explore the literature on the role of patient–provider gender concordance in mental health across diverse clinical contexts and populations.

**Method:**

We conducted a preregistered scoping review following Preferred Reporting Items for Systematic Reviews and Meta-Analyses Extension for Scoping Reviews guidelines. A comprehensive literature search was performed across MEDLINE, PsycINFO, Cochrane Library and Scopus. Study selection and screening were completed using Covidence. Two independent reviewers applied predefined criteria. Data extraction captured study characteristics, demographics, mental health conditions, interventions and outcomes. Given substantial methodological heterogeneity, findings were synthesised narratively and organised by diagnostic category.

**Results:**

This review included 33 studies representing 562 890 patients. Most studies used cohort designs to examine psychotherapy interventions in out-patient settings. Many did not specify diagnoses, although substance use and depressive disorders were common. Results were highly mixed across diagnostic categories. For general mental health, most studies found neutral or inconsistent effects. However, substance use disorder populations showed more consistent benefits, particularly for male patients in gender-matched dyads, who demonstrated improved retention, therapeutic alliance and abstinence.

**Conclusions:**

This review reveals that gender concordance effects in mental healthcare are context dependent. Whereas male patients with substance use disorders may benefit from gender matching, most evidence suggests neutral or mixed effects. Gaps in the literature include limited non-binary representation and insufficient attention to intersectional factors. Future research should employ more rigorous methods, expand beyond binary genders and investigate the mechanisms underlying observed effects.

Mental health concerns, including mood, anxiety and substance use disorders, affect a substantial portion of the population.^1^ Despite a high prevalence, mental health service utilisation remains low overall.^2^ This under-utilisation reflects a complex interplay of structural and social barriers, including limited provider availability, geographic maldistribution of services, financial constraints and social stigma. These issues, compounded by a lack of culturally or gender-affirming care, disproportionately impact marginalised groups and contribute to delays in care.

Gender significantly influences mental health service engagement. Whereas women utilise services most frequently,^2–4^ men often avoid care due to stigma and adherence to traditional masculine norms, preferring self-management or medication.^5,6^ Non-binary individuals report the highest prevalence of mental health concerns but encounter the greatest barriers, such as discrimination and a lack of gender-affirming providers.^7–9^ In addition, non-binary individuals often face greater barriers to care compared with binary transgender individuals, including lack of provider knowledge, misgendering and absence of inclusive clinical environments, which may contribute to lower engagement despite higher need.^7^

The therapeutic alliance, which is the collaborative relationship between patient and provider, is a primary predictor of positive mental health outcomes.^10^ Demographic matching between patients and providers can strengthen this alliance by improving cultural competence and trust; for instance, studies on racial concordance show that patients often experience reduced discrimination-related delays in care.^11,12^ These benefits stem from enhanced communication and shared understanding, which minimise exposure to bias or micro-aggressions. In mental health settings, such alignment facilitates disclosure and builds the foundational trust necessary for effective treatment.

Several theories support the practice of matching patients and providers based on gender, resting on broader principles from relational phenomenology suggesting that people better understand those perceived as similar.^13,14^ Developmental and social psychology suggest that gender-specific interactional styles significantly influence communication and relationship quality.^14,15^

Gender encompasses culturally influenced distinctions in personality, attitudes and behaviour that become internalised as gendered schemas, cognitive frameworks that an individual uses to interpret the world. Gender is a nuanced concept involving the interplay between spectra of femininity and masculinity and adherence to the societal expectations that come alongside these labels. Whereas biological sex and gender are distinct, gender-concordant dyads are more likely to share these worldviews, potentially strengthening the therapeutic alliance and improving outcomes. However, given the conceptual complexity of gender, it should not be assumed that patients inherently desire a provider of the same gender.

Outside of mental health, gender concordance is associated with improved care perceptions, communication and clinical outcomes in areas such as cancer screening and diabetes management, especially among female dyads.^16–18^ In mental health contexts, research shows that whereas men typically express minimal preference, women often prefer female therapists.^19,20^ However, despite these documented preferences, evidence regarding the impact of gender concordance on clinical outcomes in mental health remains limited and lacking in synthesis.

This review maps existing literature on patient–provider gender concordance in mental health, examining its impact on therapeutic dynamics, working alliance and clinical outcomes. The findings may support clinical practices prioritising thoughtful patient–provider matching to optimise care delivery and meet the unique needs of all patients seeking mental health services.

## Method

This scoping review examines the impact of patient–provider gender concordance on mental health outcomes. The review was preregistered with PROSPERO (no. CRD42024501634). The methodology was changed from a systematic to a scoping review, given the breadth of the research question. This study follows the guidelines set forth by the Preferred Reporting Items for Systematic Reviews and Meta-Analyses Extension for Scoping Reviews (PRISMA-Scr) statement.^21^

A comprehensive literature search was performed by a medical librarian on 11 January 2024 across multiple databases from inception: Ovid MEDLINE® (1946–present), Ovid PsycINFO (1967–present), Cochrane Library (Wiley) and Scopus (Elsevier). An updated search was conducted on 18 April 2025, using the same databases and search strategy. The searches included controlled vocabulary and keywords related to psychiatry, mental health services and gender concordance, without restrictions on article type or date. The searches were limited to English-language publications due to resource constraints. Reference lists and forward citations from relevant articles were also reviewed to identify additional studies. The full Ovid MEDLINE search strategy is provided in Supplementary Table 1.

A total of 4185 records from the initial database search, 1646 from the updated search and 1077 from reference lists and forward citations were imported into Covidence (https://www.covidence.org/), a systematic review-screening tool, for de-duplication, yielding 4066 unique citations. These citations were independently screened by two reviewers (J.D. and J.L.) based on titles and abstracts against predefined inclusion and exclusion criteria. Interrater agreement was initially fair (Cohen’s *κ* = 0.35), reflecting differences in interpretation of inclusion criteria, particularly around outcome definitions. Discrepancies were resolved through discussion until consensus was reached. Inclusion criteria required studies to: (a) explore gender identity concordance between providers and patients in a mental health context; (b) examine patient encounters with physicians, psychologists or counsellors in healthcare settings; (c) report outcomes such as patient adherence, disease-specific measures, mortality or patient trust and satisfaction; (d) be published in English; and (e) employ qualitative or quantitative methodologies. Exclusion criteria included: (a) studies with participants under 11 years old; (b) studies involving encounters outside healthcare settings; (c) case reports or case series; (d) studies reporting only patient gender preference without objective, measurable outcomes; and (e) studies in a language other than English. Studies were excluded if they did not report objective or measurable outcomes related to mental healthcare, such as therapeutic alliance, symptom change, treatment retention or patient-reported satisfaction, and instead focused solely on stated preferences without associated outcomes. The age cut-off of 11 years was selected to exclude early childhood populations, whose mental health presentations and therapeutic dynamics differ substantially from those of adolescents and adults. Although this threshold remains somewhat arbitrary, it reflects an attempt to balance developmental considerations with the goal of maintaining a comprehensive review. We acknowledge that the included studies may still encompass heterogeneous age groups.

**Fig. 1:**
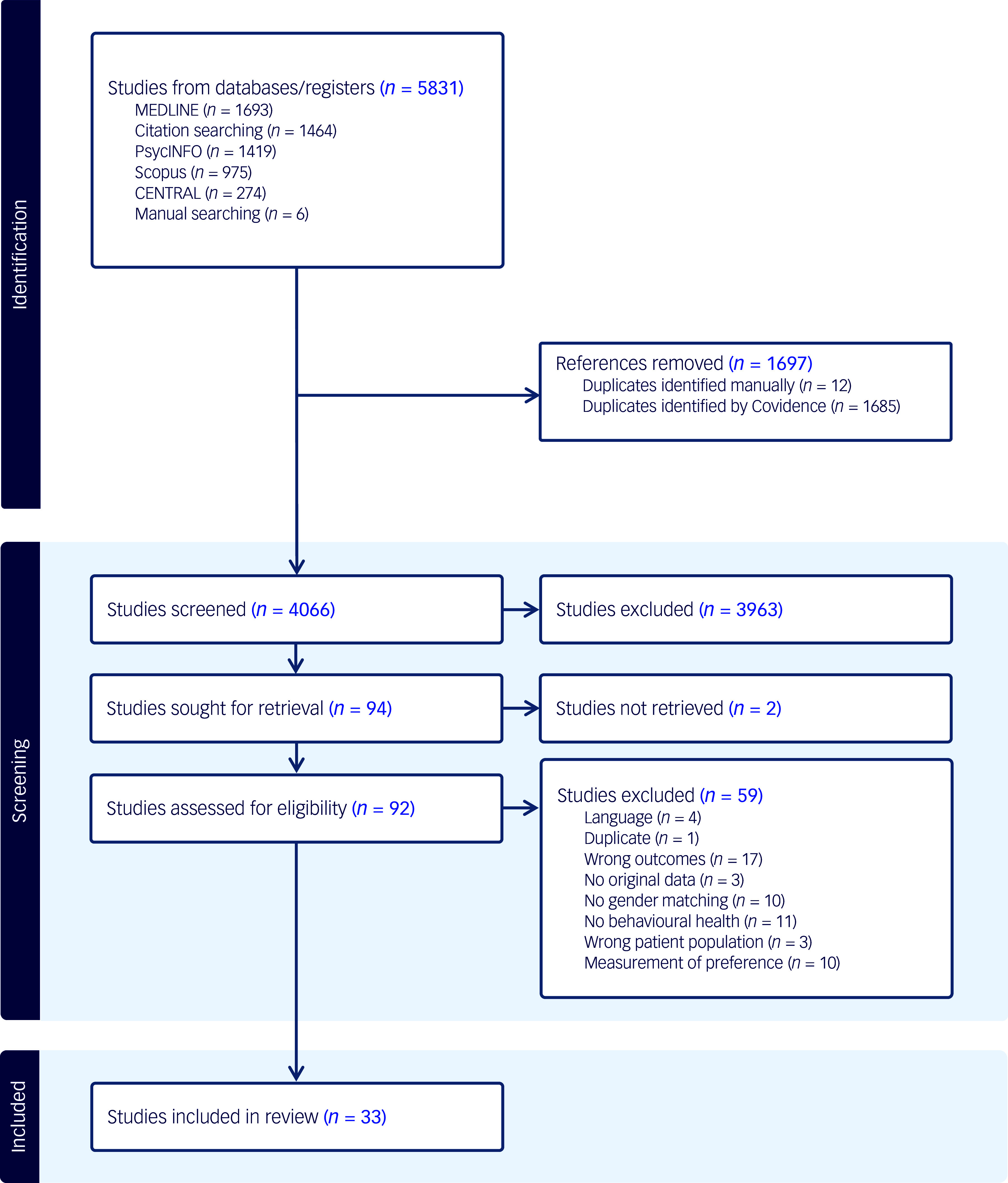
Fig. 1 long description. Preferred Reporting Items for Systematic Reviews and Meta-Analyses flow diagram outlining the study selection process. The category ‘Wrong outcomes’ is included as an umbrella reason for exclusion, encompassing scenarios in which data collected were not patient-centric behavioural health outcomes, or were outcomes presented in ways that did not allow for isolation of the effects of gender matching from other variables. The most common examples of these were qualitative outcomes, provider-centred variables and outcomes that were intersections of multiple identity concordances.

Full-text screening was similarly conducted independently by two reviewers (J.D. and J.L), with substantial interrater agreement achieved (Cohen’s *κ* = 0.71). Disagreements were again resolved through discussion and consensus. Of the 92 articles identified, 33 eligible studies met all inclusion criteria. The study selection process is outlined in the PRISMA flow diagram (Fig. 1).

Data extraction was conducted independently in Microsoft Excel (Microsoft® Excel® for Microsoft 365 MSO, version 2605 Build 16.0.20026.20140, 64-bit; https://www.microsoft.com/en-us/microsoft-365/download-office, https://app.covidence.org/) by two reviewers (J.D. and J.L.) and subsequently verified through cross-checking. Any discrepancies were resolved through discussion until full consensus was achieved. Extracted data included the following: author(s), year of publication, study sample size, patient and provider demographic characteristics (including gender identity), mental health conditions addressed, study design, healthcare setting, type of mental health intervention or encounter, measures of gender concordance, outcome variables assessed (e.g. patient adherence, symptom improvement, therapeutic alliance, patient trust and satisfaction) and key findings related to the impact of gender concordance on clinical outcomes.

Given the wide methodological heterogeneity of the studies included in the present review, a narrative synthesis approach was used to summarise and interpret the findings. This heterogeneity also precluded the possibility of conducting a meta-analysis. The included studies were organised broadly by clinical diagnosis categories (mood disorders, anxiety disorders, substance use disorders, etc.) when possible. Key findings are presented in a descriptive manner, aiming to summarise existing research. Any quantitative results were also presented narratively with respect to the clinical diagnosis. Where possible, studies were subgrouped based on the population, psychotherapy type or outcome measures, to identify trends or inconsistencies in the evidence.

## Results

This scoping review synthesised 33 studies published between 1974 and 2025, representing 562 890 patients across diverse settings and populations. Most studies (*n* = 28) were conducted in traditional out-patient or in-patient healthcare settings, whereas the remainder (*n* = 5) utilised non-traditional environments such as correctional facilities and military recruitment centres or analysed pooled data from multiple healthcare systems. In addition, studies were predominantly conducted in the USA (*n* = 24). Other represented countries include Switzerland (*n* = 2), Germany (*n* = 2), Chile (*n* = 1) and Sweden (*n* = 1). Another study used global data via smartphone application, and two others did not specify the location. Although participant age varied from adolescents to adults, most studies lacked detailed stratification, limiting the ability to report age statistics.

The studies employed various research designs, with cohort studies predominating (*n* = 21) followed by cross-sectional designs (*n* = 6). The remaining studies included secondary analyses of randomised controlled trial data (*n* = 3) and existing cohort data (*n* = 2), as well as one primary randomised controlled trial. Although all studies examined psychotherapy interventions, three also incorporated medication therapy and one evaluated broader mental health interventions.

The research addressed a wide range of mental health conditions. Over half of the studies (*n* = 17) examined general or unspecified mental health disorders whereas others focused on specific conditions including mood and anxiety disorders (*n* = 5), substance use disorders (*n* = 4), post-traumatic stress disorder (PTSD) (*n* = 2) and various other specified conditions (*n* = 5). These diagnostic categories formed the organisational framework for the subsequent narrative synthesis, with study characteristics and findings detailed in Table 1.

**Table 1:** Overview of the included studies Table 1 long description.

Author(s)	Year	Study type	Disorder studied	Number of patients	Key findings
Bhati^22^	2014	Retrospective cohort	General BH/no disorders specified	92	Female patients in matched dyads reported significantly higher therapeutic bond, working alliance, mutual affirmation and empathetic resonance than female clients in mismatched dyads. Female patients in matched dyads did not significantly outperform male patients in mismatched dyads in all measures, except mutual affirmation. Male patients in matched dyads did significantly worse than their mismatched counterparts for therapeutic bond, working alliance and empathetic resonance.
Dembo et al^23^	1983	Retrospective cohort	General BH/no disorders specified	2898	Sex matching yielded no statistically significant differences in recidivism, functional status at termination or DCMHQ measure.
Dolinsky et al^24^	1998	Prospective cohort	General BH/no disorders specified	50	A female therapist with a female patient was associated with a mutually positive assessment of their match.
Duong et al^25^	2024	Secondary analysis of a prospective cohort	General BH/no disorders specified	185	Matched dyads in which the patient gave their therapist feedback on their symptoms saw improved functional outcomes for patients. There was no significant impact of a gender match on patient trust/respect levels.
Flaskerud and Liu^26^	1991	Retrospective cohort	General BH/no disorders specified	1746	Matched male dyads saw a significantly lower drop-out. Matched male dyads did not see a significant increase in GAS over the course of the study time frame. Neither drop-out nor GAS findings showed significant gender-matching effects for female patients.
Fujino et al^27^	1994	Retrospective cohort	General BH/no disorders specified	4764	Gender matching alone had no significant impact on patient drop-out, length of treatment or GAS scores at initiation or termination.
Galuza^28^	2016	Prospective cohort	General BH/no disorders specified	7318	Gender matching yielded no significant impact on TA. There was no significant impact on treatment duration for patients in gender-matched dyads compared with their mismatched counterparts.
Hatchett and Park^29^	2004	Cohort (unspecified)	General BH/no disorders specified	245	Sex matching was not related to counselling duration.
Jerant et al^30^	2011	Prospective cohort	General BH/no disorders specified	22 440	No change from year 1 to year 2 for any concordance type was observed for the Mental Component Summary of SF-12.
Jones and Zoppel^31^	1982	Retrospective cohort	General BH/no disorders specified	160	Matched dyads saw significantly greater retention in therapy. No other sex-matching effects were seen on the Rating Scales for Therapy Outcome.
Orlinsky and Howard^32^	1976	Secondary analysis of a cohort (unspecified)	General BH/no disorders specified	118	Patients in mismatched dyads saw their therapists as significantly more demanding, more detached and less expansive, less self-possessed, less open, more self-critical and as less encouraging. Patients in matched dyads saw higher satisfaction regarding encouragement. Patients in matched dyads saw no significant change in catharsis, mastery, insight or overall experienced benefit.
Pattee and Farber^33^	2008	Cross-sectional	General BH/no disorders specified	223	Patients in female-matched dyads experienced more distress upon disclosure than male or female mismatched dyads. No significant differences were noted between dyads containing male therapists.
Persons et al^34^	1974	Cross-sectional	General BH/no disorders specified	93	Male patients in matched dyads felt that their therapists were more interested, concerned, self-disclosing and helpful with sexual identity concerns compared with those in mismatched dyads. Patients in female-matched dyads felt their therapists were more perceptive and insightful, encouraged more risk-taking, were more warm and friendly, were more helpful with sexual identity concerns, gave more honest feedback, were more self-disclosing and were more supportive than did female patients in mismatched dyads. Matched female patients had more positive take-aways from therapy than mismatched female patients.
Schmalbach et al^35^	2022	Cross-sectional	General BH/no disorders specified	1212	Patients in female-matched dyads reported higher QoL outcomes than dyads containing male therapists, and greater symptom reduction outcomes than female therapist–male patient dyads. Patients in male-matched dyads reported higher QoL outcomes than male therapist–female patient dyads, but only for psychodynamic therapy.
Staczan et al^36^	2017	Prospective cohort	General BH/no disorders specified	237	Matching on gender had no effect on patient symptomatology or therapy outcomes questionnaires. The presence of a female in a dyad improved assessment of the patient–provider relationship.
Vail^37^	1976	Prospective cohort	General BH/no disorders specified	87	Patients in matched dyads saw significantly greater drop-out than mismatched dyads.
Weinstock^38^	1999	Retrospective cohort	General BH/no disorders specified	615	Patients in matched dyads saw greater reduction regarding worry about things that they had done than those in mismatched dyads. No other subscale or question was significant.
Behn et al^39^	2018	Secondary analysis of an RCT	Mood/anxiety	547	Female patients in mismatched dyads saw significantly increased linear growth in TA compared with other dyad compositions. Matching led to no significant impact on any other patient-oriented dependent variable.
Chan et al^40^	2006	Cross-sectional	Mood/anxiety	1428	Gender concordance did not affect detection and care of BH or SUD problems in depressed patients.
Cheng and Lo^41^	2018	Secondary analysis of an RCT	Mood/anxiety	212	Client–therapist gender match overall wasbond subscale of the working alliance index strongly associated with a strong patient-reported therapeutic alliance.
Cottone et al^42^	2002	Prospective cohort	Mood/anxiety	163	The four possible client–therapist dyad groups did not differ significantly on any of the patient-oriented dependent measures.
Zlotnick et al^43^	1998	Secondary analysis of an RCT	Mood/anxiety	203	Gender matching led to no statistically significant differences in patient attrition, post-treatment HRSD or BLRI-Empathy subscale when all data were aggregated between psychotherapy and medication treatment modalities. Similar findings were observed when psychotherapy was separated from other treatments.
Semmlinger et al^44^	2025	Prospective cohort	PTSD	195	Gender matching yielded no significant differences in drop-out frequency.
Shiner et al^45^	2017	Retrospective cohort	PTSD	506 741	Male patients in mismatched dyads saw significantly greater retention than those in matched dyads. Female patients in matched dyads were not significantly different in terms of retention than their mismatched counterparts.
Fiorentine and Hillhouse^46^	1999	Prospective cohort	Substance use disorder	302	Patients in matched dyads saw significantly increased abstinence from substances. Patients in matched dyads perceived significantly greater empathy from their therapists. Gender matching had no effect on patient retention or engagement in treatment.
Gaume et al^47^	2014	Randomised controlled trial	Substance use disorder	217	Males in matched dyads who underwent brief MI for problematic alcohol use were more likely to see reductions in this than those in mismatched dyads, or the control group that underwent no intervention.
Sterling et al^48^	1998	Retrospective cohort	Substance use disorder	967	No significant effect on rates of return following initial interview was observed for male or female patients in matched or mismatched dyads.
Wintersteen et al^49^	2005	Cross-sectional	Substance use disorder	600	Patients in matched dyads saw significantly greater retention and WAI scores.
Blasko and Jeglic^50^	2016	Prospective cohort	Other (sex offenders)	202	Bond subscale of WAI for patients in mismatched dyads was significantly related to risk for sexual recidivism. No other patient-oriented outcome measures were significantly affected by gender matching.
Dailey and Claus^51^	2001	Cross-sectional	Other (victims of abuse)	8276	Male patients in mismatched dyads were significantly more likely to disclose physical or sexual abuse. Female patients in matched dyads were significantly more likely to disclose physical or sexual abuse.
Fullerton et al^52^	1990	Cohort (unspecified)	Other (personality disorders)	91	Patients matched on gender saw significantly increased GAS scores 3 months from starting therapy when their therapists had <10 years of experience. The greatest effect was seen among patients in matched dyads whose therapists had 5.0–9.5 years of experience. When looking at therapists with greater than or equal to 10 years of experience, patients in mismatched dyads saw greater improvement on GAS scores compared with those in matched dyads, although significance testing was not discussed.
Johnson and Caldwell^53^	2011	Prospective cohort	Other (MFT)	233	Patients in matched dyads reported significantly greater satisfaction than mismatched dyads.
Körlin and Wrangsjö^54^	2001	Prospective cohort	Other (music therapy)	30	Due to a small sample size and thus low power, the study was unable to detect any effects resulting from gender matching.

BH, behavioral health; DCMHQ, Denver Community Mental Health Questionnaire; GAS, global assessment scale; TA, therapeutic alliance; SF-12, Short Form 12-Question Health Survey; QoL, quality of life; RCT, randomised controlled trial; SUD, substance use disorder; HRSD, Hamilton Rating Scale for Depression; BLRI-Empathy, Barrett-Lennard Relationship Inventory Empathy Scale; PTSD, post-traumatic stress disorder; MI, motivational interviewing; WAI, working alliance inventory; MFT, marriage and family therapy.

### General mental health

Seventeen studies assessed outcomes in general mental health populations or did not specify participant conditions.^22–38^ Only two studies demonstrated consistently positive effects of gender matching for both male and female patients.^34,35^ Persons et al^34^ found that patients in matched dyads perceived therapists as more helpful, self-disclosing and supportive than did mismatched pairs. Similarly, Schmalbach et al^35^ reported that female patients in gender-matched dyads had better quality-of-life outcomes than those with male therapists, whereas male patients in matched dyads reported higher quality of life than patients in male therapist–female patient dyads. Conversely, only one study reported worse outcomes for patients in gender-matched dyads, finding significantly higher drop-out rates compared with mismatched dyads among Black patients of low socioeconomic status.^37^

Most studies reported mixed effects, with different effects by gender. In a study by Bhati,^22^ female patients in matched dyads had significantly stronger therapeutic bonds, working alliance and empathetic resonance than those in mismatched dyads, whereas patients in male-matched dyads performed worse across these measures. Orlinsky and Howard^32^ found that patients in mismatched dyads perceived therapists as more demanding, detached and less encouraging, with those in matched dyads showing higher satisfaction regarding encouragement, although no differences emerged in regard to catharsis, mastery, insight or overall benefit. Jones and Zoppel^31^ observed greater retention among patients in matched dyads, but no other outcome differences. Flaskerud and Liu^26^ found that patients in matched male dyads had lower dropout rates, although this did not translate to higher global assessment scale scores. Other studies found condition-specific effects. Weinstock^38^ reported greater worry reduction for patients in matched dyads, but no other symptom differences. Duong et al^25^ observed that patients in matched dyads with feedback showed improved functional outcomes, but no differences in trust or respect.

A subset of studies found no significant patient-centred differences between matched and unmatched dyads. For example, Jerant et al^30^ reported no change in mental health outcomes over 2 years in a large US survey of over 22 000 participants, and Hatchett and Park^29^ observed no significant variation in counselling duration between patients in matched and mismatched dyads. In a large sample of over 4000 patients, Fujino et al^27^ found that gender match alone had no significant impact on drop-out, length of treatment or global assessment scale scores at initiation or termination. Galuza^28^ reported no significant impact of gender matching on therapeutic alliance or treatment duration in over 7000 patients. Dembo et al^23^ found no statistically significant differences in recidivism, functional status at termination or mental health measures. Staczan et al^36^ observed no effect of gender matching on symptomatology or therapy outcomes, although they noted that the presence of a female in a dyad improved assessment of the patient–provider relationship.

### Mood and anxiety disorders

Five studies assessed gender-matching effects in the treatment of mood and anxiety disorders,^39–43^ three of which found no significant effects across outcome measures. Zlotnick et al^43^ reanalysed National Institute of Mental Health Treatment of Depression Collaborative Research Program data and found gender match unrelated to attrition, depression at termination or therapist empathy perceptions, whether aggregating psychotherapy and medication or examining psychotherapy separately. Cottone et al^42^ similarly found no significant differences across dyads in 163 patients with mood and anxiety disorders. Chan et al^40^ reported that gender concordance did not affect detection or care of mental health or substance use problems in over 1000 patients with depression.

Behn et al^39^ reported mixed results: female patients in mismatched dyads showed significantly increased therapeutic alliance growth compared with other compositions in a secondary analysis of randomised control trial data, although matching had no impact on other variables.^39^ However, matching led to no significant impact on any other dependent variable in this study. In contrast, Cheng and Lo^41^ found gender match strongly associated with therapeutic alliance in a secondary analysis of over 200 patients.

### PTSD

Two studies evaluated gender matching in the treatment of PTSD.^44,45^ Semmlinger et al^44^ found no statistically significant differences in drop-out rates between matched and mismatched dyads in a prospective cohort of 195 patients. In contrast, Shiner et al^45^ analysed data from over 500 000 veterans and reported that male patients in mismatched dyads saw significantly greater retention than those in matched dyads, whereas female patients in matched dyads were not significantly different in terms of retention compared with their mismatched counterparts. Notably, no study provided detailed categorisations of inciting traumatic events to determine whether trauma type modulates the effects of gender matching. Although Shiner et al^45^ included data on sexual trauma and combat exposure, other trauma categories were not explicitly addressed.

### Substance use disorders

Four studies examined the role of gender matching in the treatment of substance use disorders, including alcohol, cannabis, cocaine and opioids,^46–49^ two of which demonstrated positive effects. Wintersteen et al^49^ found that patients in matched dyads had significantly greater retention and working alliance inventory scores among 600 adolescents. Florentine and Hillhouse^46^ observed in 302 patients that matched dyads had significantly increased abstinence and perceived greater therapist empathy, although matching did not affect retention or engagement.

Gaume et al^47^ reported mixed findings in a randomised controlled trial of 217 patients: male patients in matched dyads receiving brief motivational interviewing for problematic alcohol use showed greater reductions than those in mismatched dyads or controls receiving no intervention, suggesting gender-specific benefits for male patients. Sterling et al^48^ found no significant effect on return rates for either gender among nearly 1000 patients.

Gender-specific effects were most pronounced among male patients with substance use disorders. Two studies reported significantly improved outcomes for male patients in gender-matched dyads,^47,49^ whereas one found no effect.^48^ For female patients, results were more mixed, with one study showing benefits of gender matching^46^ and two finding no differences.^48,49^

### Other mental health conditions and unique modalities

Five studies addressed a variety of other mental health conditions or examined unique treatment modalities that do not lend themselves well to inclusion in other categories, with findings varying widely depending on the population and outcomes assessed.^50–54^ As such, these five studies will be discussed individually.

A study in marriage and family therapy by Johnson and Caldwell^53^ found that gender matching had no effect on provider confidence or patient satisfaction. Another study by Fullerton et al,^52^ examining patients with personality disorders, reported better global assessment scale scores for patients in matched dyads.

Blasko and Jeglic^50^ studied over 200 incarcerated male sexual offenders and found that the bond subscale of the working alliance index for patients in mismatched dyads was significantly related to risk for sexual recidivism, although no other outcome measures were significantly affected by gender matching.

Dailey and Claus^51^ examined physical or sexual abuse disclosure and found gender-specific effects, with improved disclosure for both male and female patients matched with female providers. Finally, Körlin and Wrangsjö^54^ reported that their small-sample study of 30 patients undergoing guided imagery and music therapy lacked sufficient power to detect meaningful differences based on gender matching.

## Discussion

This review reveals substantial heterogeneity in the evidence surrounding patient–provider gender concordance in mental health outcomes. Whereas most studies found no significant or consistent association, a subset suggested that gender matching may have beneficial effects in specific contexts. However, even when statistically significant effects were detected, most were typically small in magnitude. Among studies examining general mental health populations, findings ranged from beneficial to neutral to potentially detrimental. For mood and anxiety disorders the evidence was predominantly neutral, with only isolated positive findings. In substance use disorders, male patients appeared to benefit more consistently from gender matching whereas effects for female patients were less consistent. Several studies identified what has been termed a ‘female effect’, where female therapists appeared to develop stronger therapeutic alliances regardless of patient gender. Female therapists have been theorised to possess stronger relationship-building capabilities due to both socialisation towards communal orientations and cultural emphasis on interpersonal connection.^55^ This female effect may also reflect broader societal expectations that position women as more emotionally attuned or nurturing, creating a self-fulfilling prophecy where patients expect greater warmth from female providers. Understanding whether this effect reflects true differences in relational skill or perception bias remains an important area for future research.

### Practice implications

These findings suggest that, although patient preferences should be respected when feasible, clinical practice should prioritise provider competence, training and the ability to establish strong therapeutic alliances over gender matching alone. In settings where gender matching is not possible or would result in treatment delays, providers should focus on developing culturally responsive therapeutic skills. Healthcare systems should invest in training all therapists in gender-responsive care approaches that enable them to work effectively with patients of all genders, rather than relying primarily on matching strategies.

The lack of strong or consistent evidence supporting gender matching as a universally beneficial strategy in psychotherapy suggests that the impact of gender concordance may vary depending on the clinical context and individual preferences. In some cases, matching gender may improve the therapeutic alliance and retention, particularly in stigmatised or gendered health conditions, whereas in others it may offer no added benefit and attempting gender matching may delay care. For example, one study among Black patients of low socioeconomic status found worse outcomes in gender-concordant dyads, suggesting that cultural or racial concordance, or provider attitudes and biases, may intersect with gender in complex ways.^37^ The influence of provider attitudes or biases is particularly relevant when interpreting this finding, because research has extensively documented that Black patients with substance use disorder are less likely to receive standard of care compared with White patients.^56^

### Future research

Whereas gender matching may play a meaningful role in certain contexts, evidence does not support its widespread implementation as a universal strategy. Future research should explore other components of identity, including gender identity beyond the binary and intersecting factors such as race, class and provider training. Many studies examining gender matching suffer from design limitations, such as examining it only as a *post hoc* hypothesis or small sample sizes. Randomised controlled trials and qualitative investigations are needed to better understand when, for whom and why gender concordance may matter in mental health.

Research should also investigate alternative explanations for observed gender effects, such as whether women systematically rate therapeutic relationships more positively due to socialisation factors rather than actual differences in alliance quality. Studies incorporating both self-reported and observer-rated measures of alliance could help disentangle rating biases from genuine therapeutic effects. Additionally, investigation into the mechanisms underlying the female effect observed in multiple studies may inform training programmes designed to enhance all therapists’ relational competencies.

Future research should also examine both condition- and outcome-specific effects more systematically, because this review suggests that gender-matching effects may vary by diagnosis (more beneficial in substance use disorders than in mood/anxiety disorders), by specific outcomes measured (alliance versus symptom reduction versus retention) and by patient gender (potentially more beneficial for male patients with substance use disorders). Additionally, future research regarding the impacts of gender matching by traumatic exposures in PTSD would be beneficial, because the type of trauma probably influences patient needs and therapeutic dynamics.

### Limitations

This review has several important limitations. As a scoping review, we did not conduct formal quality assessment or risk of bias evaluation because our focus was on describing how gender concordance impacted mental health outcomes rather than on evaluating the efficacy of specific interventions. Despite no formal quality assessments, the strength and reliability of evidence varied considerably across studies due to differences in sample sizes (ranging from 30 to over 500 000 participants), methodologies and settings. Several studies dated back over 50 years, reflecting older methodological standards. Considerable heterogeneity in outcome definitions precluded meta-analysis and limited generalisability. In addition, most studies relied on a binary definition of gender and did not assess or report on gender identity beyond male or female. Only two patients and no providers explicitly identified as non-binary, making it impossible to evaluate matching for gender-diverse individuals. Because most data collection did not distinguish transgender status, no conclusions can be drawn regarding how gender matching affects behavioural health outcomes for transgender patients. This remains an important area for future study.

Geographic concentration posed another limitation, because nearly all studies were USA-based, limiting generalisability to other cultural and healthcare contexts. Few studies examined provider-level factors such as experience or training that might mediate gender-matching effects. For example, one study^52^ suggested that the benefit of gender concordance diminished with increasing provider experience, highlighting the potential role of clinical skill over demographic concordance. Furthermore, most studies were observational, raising concerns about unmeasured confounding, and only three studies discussed medication management in addition to psychotherapy. Finally, the complex nature of identity presents inherent methodological challenges for patient–provider-matching research. Identity encompasses multiple intersecting variables (race, gender, socioeconomic status, language and contextual factors) that bidirectionally influence care delivery and outcomes. These interactions create substantial confounding that limits the detection of meaningful associations. Additionally, the diagnostic and management complexity of mental health conditions, influenced by individual variability and biological, social and societal interactions, further complicates interpretation of gender-matching effects. Finally, given the paucity of clear data in the literature focusing on traumatic index events or experiences in patients with PTSD, we are unable to comment on the effects this may have on gender matching in this specific population.

This review highlights the complexity and context-specific nature of gender matching in mental healthcare. Although most studies found no significant effect, certain subgroups, particularly male patients with substance use disorders, appeared to benefit from gender concordance with their providers. However, the current literature is limited in both scope and inclusivity. The overwhelming focus on binary gender categories, the underrepresentation of non-binary individuals and the predominance of studies conducted in the USA all point to important gaps in the evidence base. Because perceptions of gender and mental health vary by culture, future research should expand to include more diverse populations and international settings. Additionally, studies should intentionally include and analyse the experiences of non-binary patients and providers to ensure that mental healthcare becomes more inclusive, equitable and responsive to the needs of all individuals.

## Supporting information

10.1192/bjo.2026.12036.sm001 Lanni et al. supplementary material Lanni et al. supplementary material

## Data Availability

Data availability is not applicable to this article because no new data were created or analysed in this study.

## Supplementary material

The supplementary material is available online at https://doi.org/10.1192/bjo.2026.12036

## Fig. 1 Long description

The flowchart begins with the identification stage, where studies are sourced from various databases and registers, including MEDLINE, PsycINFO, Scopus, CENTRAL, and manual searches, totaling 5831 studies. References removed due to duplicates identified manually and by Covidence amount to 1697. In the screening stage, 4066 studies are screened, with 3963 studies excluded. Of the remaining, 94 studies are sought for retrieval, with 2 studies not retrieved. 92 studies are assessed for eligibility, and 59 studies are excluded for reasons such as language, duplicates, wrong outcomes, no original data, no gender matching, no behavioral health focus, wrong patient population, and measurement of preference. Finally, 33 studies are included in the review.

Navigate back to Fig. 1.

## Table 1 Long description

The table presents an overview of studies on various mental health conditions. It includes columns for author names, year of study, study type, disorder studied, number of patients, and key findings. The studies cover a range of mental health disorders, including general and unspecified disorders, mood and anxiety disorders, substance use disorders, PTSD, and other specified conditions. Each row details specific studies, their methodologies, and notable outcomes, providing a comprehensive summary of research in the field.

Navigate back to Table 1.
