# Supplementary material for: Impact of patient–provider gender concordance on mental health outcomes: scoping review
Source: BJPsych Open. 2026 Jul 17;12(4):e189. doi: 10.1192/bjo.2026.12036 (PMC13419668; doi:10.1192/bjo.2026.12036)
Supplement: Lanni et al. supplementary material [file S2056472426120365sup001.docx]

**Supplementary Table 1:** Ovid Medline Search Strategy

| 1 | Psychiatrists/ or Therapeutic Alliance/ or exp Psychiatry/ or exp Psychology/ or Psychotherapy/ or (psychiatr* or therapeutic alliance* or psycholog* or psychotherap* or psychoanalys*).ti,ab,kf. |
| --- | --- |
| 2 | Professional-Patient Relations/ or Physician-Patient Relations/ or Counselors/ or Counseling/ or Physicians, Primary Care/ or Primary Health Care/ or Physicians, Family/ or General Practitioners/ or (((doctor* or physician* or professional) adj3 patient* adj3 relation*) or counsel?or* or therapist* or counsel?ing or (primary adj2 (care or healthcare)) or ((family or general) adj2 (doctor* or physician* or practitioner*))).ti,ab,kf. |
| 3 | Mental Health Services/ or Mental Health/ or Behavioral Medicine/ or (((behavioral or behavioural or mental) adj2 (care or health* or service*)) or ((behavioral or behavioural) adj2 medicine)).ti,ab,kf. |
| 4 | 2 and 3 |
| 5 | 1 or 4 |
| 6 | (gender adj3 (concordan* or match* or preference*)).ti,ab,kf. |
| 7 | 5 and 6 |
